# A case report of asymptomatic aortic thrombosis incidentally detected by computed tomography in apparently healthy subject with a history of cancer surgery

**DOI:** 10.1186/s12959-016-0090-4

**Published:** 2016-08-02

**Authors:** Tomonori Sugiura, Yasuaki Dohi, Sumiyo Yamashita, Shunsuke Murai, Nobuyuki Ohte

**Affiliations:** 1 Department of Cardio-Renal Medicine and Hypertension, Nagoya City University Graduate School of Medical Sciences, Kawasumi 1, Mizuho-cho, Mizuho-ku, Nagoya, 467-8601 Japan; 2 Department of Internal Medicine, Faculty of Rehabilitation Science, Nagoya Gakuin University, Nagoya, Japan

**Keywords:** Aortic thrombosis, Incidental, Computed tomography, Pharmacotherapy

## Abstract

**Background:**

Aortic thrombosis is a rare disease and only a few cases of the disease, especially associated with chemotherapy for malignant diseases and/or blood diseases, have been previously reported. Although Virchow’s triad for thrombogenesis, namely hypercoagulability, blood flow stasis, and vessel wall injury, is the major factor promoting the formation of thrombosis, the detailed mechanism of the disease has not been well established.

**Case presentation:**

We report a case of aortic thrombosis incidentally detected by computed tomography and then regressed by pharmacotherapy using warfarin. This case is an apparently healthy man in a postoperative state after lung cancer surgery with decreased protein-C activity.

**Conclusions:**

A case of aortic thrombosis without an obvious abnormality of the aorta was incidentally identified. A few cases of aortic thrombosis in healthy aortas have been reported to be associated with chemotherapy or blood diseases, however our present case did not had such a background. Although the detailed mechanism remains to be elucidated, this case suggests that aortic thrombosis can develop in apparently healthy subjects with a history of cancer surgery.

## Background

Aortic thrombosis is a rare disease and only a few cases have been previously reported [1–3]. Malignant diseases and/or blood diseases, such as thrombocytosis and chemotherapy-related hematological disorders, have been identified as one of the causes of aortic thrombosis in these previous reports [1–5]. While Virchow’s triad for thrombogenesis, namely hypercoagulability, blood flow stasis, and vessel wall injury, is the major factor accelerating the formation of thrombus in the aortic wall, the detailed mechanism of aortic thrombosis has not been fully elucidated. Moreover, its therapeutic strategy is still controversial [6].

Previously, we reported a case of multiple aortic thrombosis in a patient with malignant lymphoma complicated by splenic infarction, and successfully treated by non-invasive pharmacotherapy [7]. Since then, we have identified another case of aortic thrombosis which was incidentally detected by computed tomography (CT), when performed for follow-up of primary diseases. Unlike our previous report, the contribution of malignant disease and chemotherapy was not suggested in this case. Here, we report a case of aortic thrombosis with background of surgery for lung cancer 1.5 years ago and no signature of recurrence.

## Case presentation

**Fig. 1:**
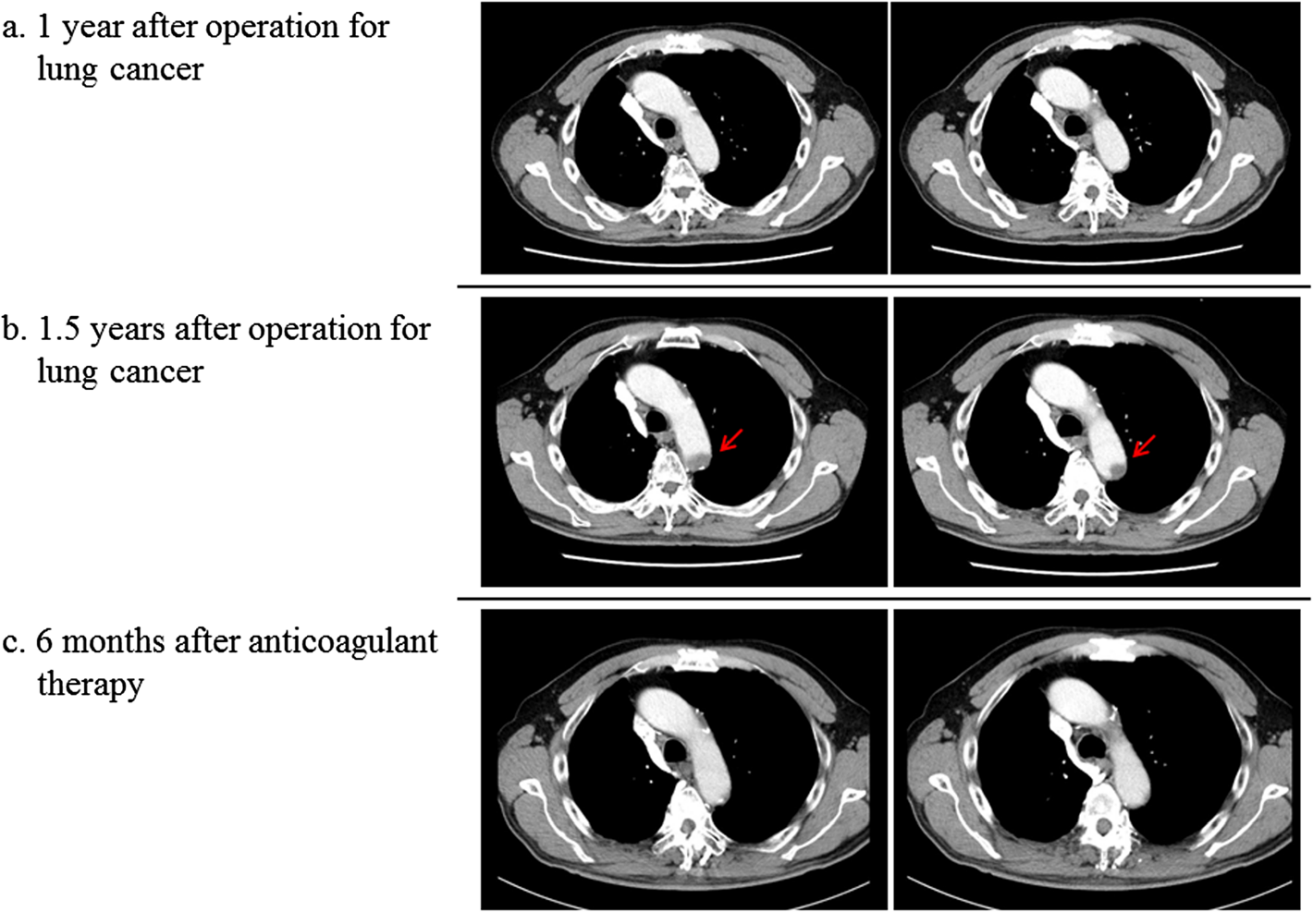
Contrast-enhanced computed tomography (CT) of the chest undertaken 1 years after surgery for lung cancer (**a**) Contrast-enhanced CT of the chest 1.5 years after surgery for lung cancer. An aortic thrombus was incidentally detected in the distal aortic arch (**b**) Contrast-enhanced CT of the chest 2 years after surgery for lung cancer. The thrombus in the distal aortic arch has disappeared after 6 months of anticoagulant therapy without an event of symptomatic distal embolism (**c**) *Arrows* indicate thrombosis

A 75-year-old Japanese man who had no history of thrombosis or thromboembolism underwent resection of his right upper lung lobe for adenocarcinoma of localized adenocarcinoma without distant metastasis, 2 years previously. Chemotherapy and radiation therapy were not performed. After the operation, periodic follow-up CT was undertaken every 6 months and fluorodeoxyglucose-positron emission tomography (FDG-PET) was performed after 1 year of operation in order to check for recurrence of lung cancer, and the results showed no recurrence or distant metastasis and no abnormality of the thoracic aorta or unexpected mural thrombus (Fig. 1a). However, an aortic thrombus was incidentally detected in the descending aortic arch 1.5 years after the primary operation (Fig. 1b). At that time, the patient was apparently healthy, with a body temperature of 36.2 °C, blood pressure of 122/68 mmHg, heart rate of 76 bpm, and peripheral oxygen saturation of 96 % in room air. Laboratory data showed slightly elevated D-dimer, decreased plasma protein-C activity, and normal liver function and lipid profile (Table 1). Lupus anticoagulant or anticardiolipin antibody was not detected and the patient stopped smoking at 60-year-old (Brinkman index 800). Although the cause and significance of decreased protein-C activity was not fully understood, we started anticoagulant therapy for aortic thrombosis using warfarin. Follow-up CT showed the aortic thrombus was gradually regressing and was eliminated completely after 6 months of anticoagulant therapy without major complications (Fig. 1c). Then, anticoagulant therapy was terminated without recurrence of aortic thrombus and plasma protein-C activity was recovered within normal range (72 %).

**Table 1:** Characteristics of the case

Variable	Patient value	Reference range
White Blood Cell (/μL)	5400	3600–9600
Hemoglobin (g/dl)	14.9	13.2–17.2
Platelet (/10^−4^ μL)	12.3	14.8–33.9
CK (U/L)	150	62–287
AST (U/L)	18	13–33
ALT (U/L)	17	6–30
LDH (U/L)	171	119–223
Creatinine (mg/dl)	0.89	0.60–1.10
Causal blood glucose (mg/dl)	107	70–139
Total-cholesterol (mg/dl)	165	128–219
HDL-cholesterol (mg/dl)	43	40–96
CRP (mg/dl)	0.06	<0.3
BNP (pg/ml)	31.1	< 18.4
APTT (%)	116.1	76.0–130.0
PT (%)	91.6	70.0–130.0
HbA1c (%)	6.1	4.6–6.2
D-dimer (μg/ml)	1.5	<0.5
Protein C activity (%)	45	64–146
Protein C level (%)	39	70–150
Total protein S antigen (%)	84	60–150
Free Protein S antigen (%)	91	65–135
Antithrombin III (mg/dl)	25.4	22.6–33.5
Lupus anticoagulant (second)	Undetected	-
Anticardiolipin antibody	Undetected	-
Anticardiolipin β2GPI antibodies	Undetected	-

*CK* creatine kinase, *AST* aspartate aminotransferase, *ALT* alanine aminotransferase, *LDH* lactate dehydrogenase, *HDL* high-density lipoprotein, *CRP* C reactive protein, *BNP* brain natriuretic peptide, *APTT* activated partial thromboplastin time, *PT* prothrombin time, β*2GPI* β2 antiglycoprotein I

Aortic thrombosis is a rare disease which can cause distal embolism, but can also be detected incidentally in asymptomatic cases [1, 2]. Although aortic mural thrombus associated with abnormal aortic disease, such as aortic aneurysm and aortic dissection, is often seen, a thrombus in an apparently healthy aorta is very rare, since the aortic blood flow is too fast for clotting and the formation of a growing thrombus [1, 2]. Virchow’s triad for thrombogenesis, featuring hypercoagulability, blood flow stasis, and vessel wall injury, is known to be important in thrombus formation, however the concept is generally adopted for thrombosis in veins and/or small arteries [6]. On the other hand, thromboembolism complicated with atrial fibrillation is well-known, but arrhythmia cannot be the etiology of local thrombus of the aorta. Thus, the detailed mechanism of the etiology of aortic thrombosis is not well understood [1, 2].

In the present report, we describe a case of aortic thrombosis which was seen in an apparently healthy man in a postoperative state after lung cancer surgery with decreased protein-C activity. Although hereditary protein-C deficiency is well known to be associated with recurrent thrombosis [8, 9], this patient and his family did not have episodes of thrombosis. Moreover, the protein-C activity was recovered within normal range after termination of anticoagulant therapy. Protein-C deficiency is often seen in the acute phase of thrombosis and in patients undergoing anticoagulation therapy, since protein-C and protein-S are vitamin K-dependent glycoproteins that are massively lost in such conditions [8, 9]. Thus, the contribution of protein-C deficiency to the development of aortic thrombosis, while possible, is not clear. Present case is in contrast to several recent reports of aortic thrombosis which were associated with malignant disease, hematological disorders, and chemotherapy-related thrombosis, especially in relation to cisplatin-based chemotherapy [4, 5]. Actually, the background of our previously reported case of aortic thrombosis included malignant lymphoma and related chemotherapy [6].

The therapeutic strategy for aortic thrombosis is controversial, however, the main strategy is conservative pharmacotherapy [1–3]. There is no definitive evidence as to the antithrombotic therapy to be selected or the appropriate duration of such treatment in the case of aortic thrombosis. Therefore, we chose warfarin therapy for 6 months in accordance with the standard therapy in the case of venous thrombosis. After termination of the warfarin therapy, the recurrence of aortic thrombosis has not been confirmed more than 6 months. Another strategy is surgical therapy, which is particularly useful for symptomatic cases with distal thromboembolism, and combined therapy may also be selected occasionally [1–3]. We selected conservative pharmacotherapy since signs of distal thromboembolism were not evident. However, recently the technique of aortic stent graft treatment has been dramatically improved, allowing an alternative treatment choice for aortic thrombosis, especially in emergency cases or in unfavorable conditions [10].

## Conclusions

We identified a case of aortic thrombosis without an abnormality of the aorta. A few cases of aortic thrombosis in healthy aortas have been reported to be associated with chemotherapy or blood diseases, however our present case did not have such a background. Although the detailed mechanism remains to be elucidated, this case suggest that aortic thrombosis can develop in apparently healthy subjects with a history of cancer surgery.

## Abbreviations

APTE, acute pulmonary thromboembolism; CT, computed tomography; FDG-PET, fluorodeoxyglucose-positron emission tomography
